# A reduced concentration femoral nerve block is effective for perioperative pain control following ACL reconstruction: a retrospective review

**DOI:** 10.1007/s00402-021-04221-3

**Published:** 2021-10-21

**Authors:** Lukas N. Muench, Megan Wolf, Cameron Kia, Daniel P. Berthold, Mark P. Cote, Adam Fischler, Robert A. Arciero, Cory Edgar

**Affiliations:** 1 Department of Orthopaedic Surgery, UConn Health, Farmington, CT USA; 2 Department of Orthopaedic Sports Medicine, Technical University of Munich, Munich, Germany; 3 Department of Anesthesiology, UConn Health, Farmington, CT USA

**Keywords:** Femoral nerve block, Anterior cruciate ligament reconstruction, ACL, Quadriceps motor function, Local anesthetic

## Abstract

**Introduction:**

Femoral nerve block (FNB) is a routinely used regional analgesic technique for anterior cruciate ligament (ACL) reconstruction. One method to balance the analgesic effect and functional impairment of FNBs may be to control the concentration of local anesthetics utilized for the block.

**Materials and methods:**

Retrospective chart review was performed on 390 consecutive patients who underwent ACL reconstruction between June 2014 and May 2017. Patients were divided into those who received a standard (0.5%-bupivacaine) or low (0.1–0.125%-bupivacaine) concentration single-shot FNB performed with ultrasound guidance. Maximum postoperative VAS, Post-Anaesthesia Care Unit (PACU) time prior to discharge, need for additional ‘rescue’ block, and intravenous postoperative narcotic requirements were recorded.

**Results:**

A total of 268 patients (28.4 ± 11.9 years) were included for final analysis, with 72 patients in the low-concentration FNB group and 196 patients receiving the standard concentration. There were no differences in the maximum postoperative VAS between the low (6.4 ± 2.5) and standard (5.7 ± 2.9) concentration groups (*P* = 0.08). Similarly, the time from PACU arrival to discharge was not different between groups (*P* = 0.64). A sciatic rescue block was needed in 22% of patients with standard-dose FNB compared to 30% of patients receiving the low-concentration FNB (*P* = 0.20). Patients with a hamstring autograft harvest were more likely to undergo a postoperative sciatic rescue block compared to a bone-patellar tendon autograft (*P* = 0.005), regardless of preoperative block concentration. Quadriceps activation was preserved with low-concentration blocks.

**Conclusions:**

Using 1/5th to 1/4th the standard local anesthetic concentration for preoperative femoral nerve block in ACL reconstruction did not significantly differ in peri-operative outcomes, PACU time, need for rescue blockade, or additional immediate opioid requirements.

**Level of Evidence:**

III.

## Introduction

Anterior cruciate ligament (ACL) tears are among the most common orthopaedic injuries, with an increasing number of reconstructive surgeries performed annually [15, 19]. As ACL reconstruction is almost exclusively performed in the ambulatory setting, perioperative analgesia has been shown to be an essential component for a timely discharge to home, with good safety and adequate short-term pain control [3, 14, 20, 23]. This has also been implicated to affect the longer-term postoperative course, with inferior patient-reported outcomes and strength deficits depending on the regional anesthetic of choice [14, 21]. Although multiple local and peripheral nerve block modalities have been investigated, there is no current consensus for the pain management of these patients [21].

Femoral nerve block (FNB) is a routinely used regional analgesic technique for ACL reconstruction, with multiple studies demonstrating its effectiveness in the early postoperative period, including earlier Post-Anesthesia Care Unit (PACU) discharge [7, 17, 20, 23]. Previous randomized control trials have found that a single-shot FNB resulted in significantly reduced pain and opioid consumption compared to placebo [8, 17, 21].

However, along with quadriceps weakness FNBs and their effect on postoperative muscle recruitment have also been linked to delayed return to sport following ACL reconstruction, especially in a young patient cohort [14, 18]. Luo et al. found that pediatric and adolescent patients who received single-shot FNBs had significant deficits in isokinetic testing of knee extension and flexion, along with a fourfold likelihood to not return to sport at six months compared to patients who received no regional anesthetic [14]. These findings remain a major concern, as rehabilitation strategies following ACL reconstruction are moving towards more aggressive and earlier integration of strength training, to promote successful return to pre-injury activities [2].

While other modalities such adductor canal blocks [18] and local infiltration have been suggested, the variability in efficacy [10, 24] and technique [12, 16] of these treatments warrant further solutions. One method to balance the analgesic effect and functional impairment of single-shot FNBs, may be to control the concentration of local anesthetics utilized for the block [4, 22]. Thus, the purpose of the study was to compare the immediate perioperative outcomes of patients undergoing arthroscopic ACL reconstruction who received a standard 0.5% bupivacaine single-shot femoral nerve block to those who have received a single-shot with 0.1–0.125% bupivacaine concentration. The authors hypothesized that patients who preoperatively received a lower concentration of anesthetic would have no significant difference in perioperative pain scores and similar requirement for additional rescue blocks compared to the standard local anesthetic concentration.

## Materials and methods

### Study design

**Fig. 1:**
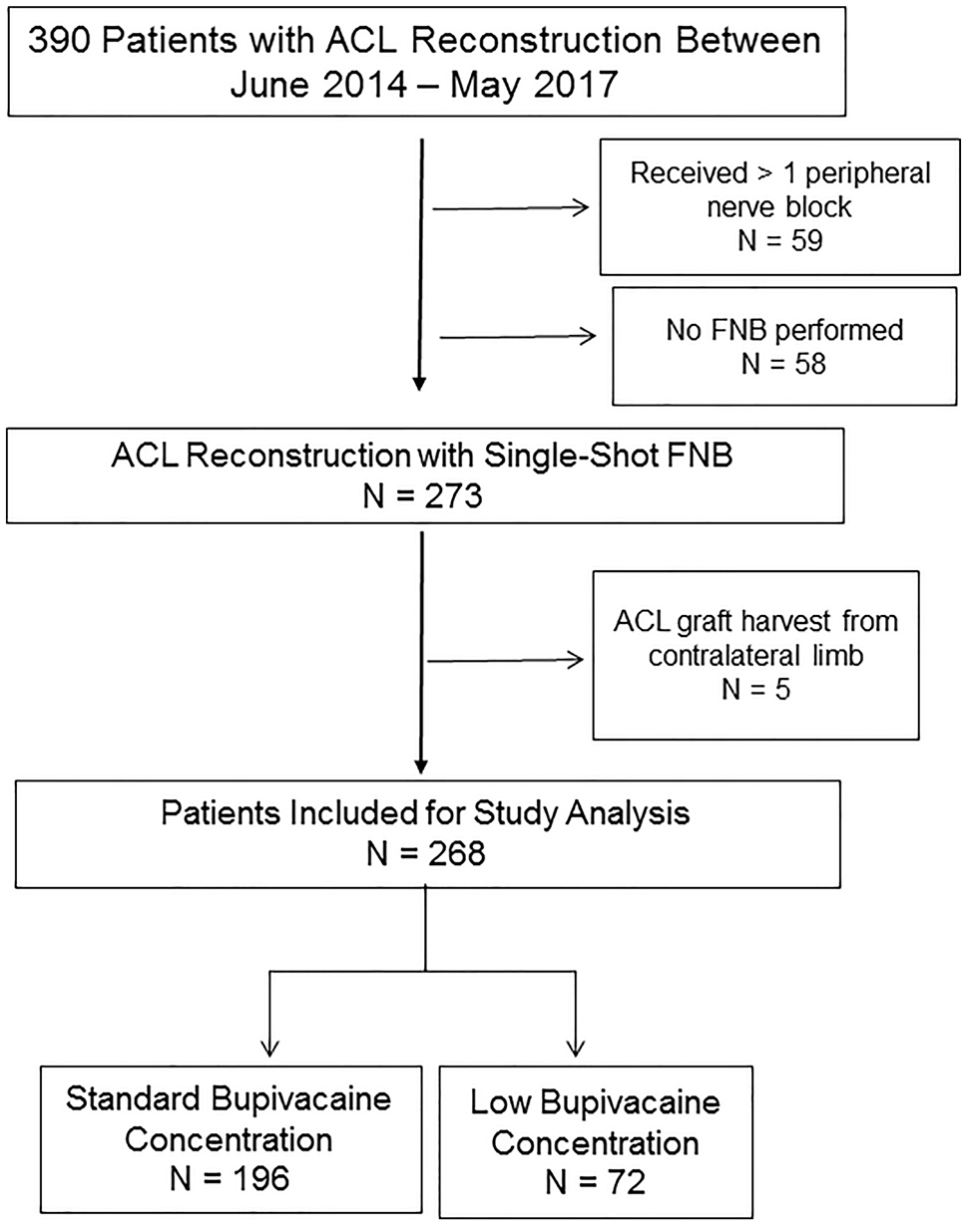
Flow diagram demonstrating patients included for study analysis

A retrospective chart review was performed at a single institution between June 2014 and May 2017 to identify all patients who underwent arthroscopic ACL reconstruction. Institutional review board approval was obtained prior to the initiation of the study (IRB# XXX-XX-X). Inclusion criteria included any patient who underwent either primary or revision ACL reconstruction with a preoperative femoral nerve block. Any patients who underwent contralateral limb autograft harvest or had multiple preoperative nerve blocks were excluded from the study (Fig. 1). Patients were then grouped based on the concentration of bupivacaine used for the single-shot femoral nerve block; either with standard (0.5%) or low (0.1–0.125%) concentration, according to the anesthesiologist’s preference. There was no blinding of the used concentration to the anesthesia team. Patient demographics including age, sex, body mass index (BMI), and history of narcotic use between the two groups were recorded.

### Perioperative outcomes measures

Several perioperative outcomes were used to compare between groups. This included total PACU time (arrival to discharge), requirement for a rescue block (femoral or sciatic), need for intravenous narcotics in the PACU, and maximum visual analogue scale (VAS) score. Criteria for PACU discharge were that the patient was able to mobilize safely with crutches, was pain controlled with a VAS score of less than 5, and was saturating appropriately on room air. Rescue blocks were determined with discussion with the patient, surgeon and anesthesiologist with consent obtained preoperatively, prior to induction of anesthesia. If the patients’ pain was not deemed suitable for discharge after 30 min of attempted pain control, a rescue block was offered to the patient. Patients were asked to provide both the maximum and minimum VAS score prior to discharge, and the maximum score was used for analysis. Tourniquet time, type of graft, and the influence of revision surgery were also compared between groups.

### Femoral nerve block

All femoral nerve blocks were performed with ultrasound guidance by anesthesiologists experienced in administering regional anesthesia. All blocks were performed in the preoperative area prior to induction of general anesthesia. Using the same previously described single-shot technique [18], the femoral nerve was located distal to the inguinal ligament under ultrasound guidance in each patient. Following sterile skin preparation, a 22- or 21-gauge needle (Stimuplex A, B. Braun Medical Inc., PA, USA) was used to administer 25–30 mL of either 0.5% or 0.1–0.125% bupivacaine with epinephrine 1:200,000 lateral to the femoral nerve in an in-plane technique. Muscle stimulators were not used. Normal saline was used for bupivacaine dilution. In addition to observing perineural spread of local anesthetic, success of the block was determined by the anesthesia team assessing tactile sensation along with the femoral nerve distribution.

### Statistical analysis

An a priori power analysis was performed using G*Power (Erdfelder, Faul, Buchner, Lang, HHU Düsseldorf, Düsseldorf, Germany) to determine detectable differences in VAS score given estimated standard deviations [6]. Assuming a common standard deviation of 1.0, a sample size of 156 patients would provide 80% power to detect a 0.5 difference in VAS score at an α level of 0.05.

Descriptive statistics included mean and standard deviation (SD), as well as frequency and proportions where appropriate. Differences in continuous variables between low versus standard concentration femoral nerve block groups were compared using the independent *t* test. Categorical differences between the low versus standard concentration femoral nerve block groups were compared using the chi-square test or Fisher’s exact when expected call values were less than 5. Following univariate analysis, factors that were found to trend towards significant predictors of needing a postoperative rescue block were placed in a multi-variate analysis for odds ratio. The results of inferential statistics were reported with 95% confidence intervals. All statistical analysis was performed using Stata 15.2 software (StataCorp 2017. Stata Statistical Software: Release 15).

## Results

### Subjects

Of the 390 patients found from the initial chart review, 268 patients met the final inclusion criteria. Mean age of all patients was 28.4 ± 11.9 years with 148 males and 120 females included. The standard concentration (0.5%) was given to 196 patients while 72 patients received the low concentration (0.1–0.125%) dose. Majority of surgeries were primary ACL reconstruction (85%), with bone-patellar bone autograft being the most commonly used graft. The mean dose of intraoperative fentanyl given to the standard concentration FNB patients was 170mcg compared to 166 mcg in the low concentration FNB group (*P* = 0.71). There were no differences in patient demographics, tourniquet time, graft type, or concomitant procedures between the two groups (Table 1).

**Table 1:** Patient demographics and operative type between treatment groups

	Standard concentration bupivacaine dose		Low concentration bupivacaine dose		*P* value
	(*n* = 196)		(*n* = 72)		
		%		%	
*Sex (n)*					
Male	113	58%	35	48%	0.19
Female	83	42%	37	52%	
*Mean age ± SD*	28.7 ± 11.8		28.0 ± 11.5		0.64
*BMI ± SD*	27 ± 21.4		27 ± 5.2		0.59
*Surgery type (n)*					
Primary	167	86%	63	88%	> 0.05
Revision	29	14%	9	12%	
*Tourniquet used*	136	70%	58	80%	0.08
*Tourniquet time (minutes) graft type (n)*	86 ± 23.2		92.0 ± 20.2		0.09
BTB autograft	71	36%	26	36%	> 0.05
Hamstring autograft	58	30%	20	28%	
Quadriceps autograft	12	6%	1	2%	
Hybrid	7	4%	10	13%	
Allograft	48	24%	15	21%	
*Concomitant procedure*					
Additional ligament reconstruction or repair	8	11%	32	16%	> 0.05
Meniscal repair or meniscectomy	55	76%	135	69%	

Abbreviations: *BMI *body mass index, *SD *standard deviation, *BTB *bone-patellar tendon-bone

### Perioperative outcomes

There was no significant difference in maximum postoperative VAS scores for patients who received standard (5.7 ± 2.9) or low (6.2 ± 2.4) concentration femoral nerve blocks (*P* = 0.08). Additionally, there was no significant difference in PACU time when comparing patients who underwent a low (115.8 ± 42.9 min) or standard (117.6 ± 36.2 min) concentration FNB (*P* = 0.64). Similarly, no significant difference was found in the rate of postoperative rescue block between the standard (22%; *N* = 43) and low (30%; *N* = 22) concentration groups (P = 0.20). Of these patients, a repeat FNB was only needed in 1 patient of the low compared to 3 patients of the standard concentration group. Sixty-one patients required a postoperative sciatic rescue block (40 in the standard versus 21 in the low concentration group). The need for postoperative intravenous narcotic was also not found to be different between groups (*P* = 0.53) (Table 2).

**Table 2:** Perioperative outcomes between femoral nerve block concentrations

	Standard concentration FNB	Low concentration FNB	*P*-value
Maximum postop VAS (± SD)	5.7 (± 2.9)	6.2 (± 2.4)	0.08
Total PACU time in min (± SD)	117 (± 35.8)	115 (± 42.9)	0.64
Postoperative rescue block *N* (%)	43 (22%)	22 (30%)	0.20
Intravenous postoperative pain medication *N* (%)	77 (35%)	25 (39%)	0.53

Abbreviations: *FNB *femoral nerve block, *VAS *visual analogue scale, *PACU *post-anesthesia care unit, *SD *standard deviation

**Fig. 2:**
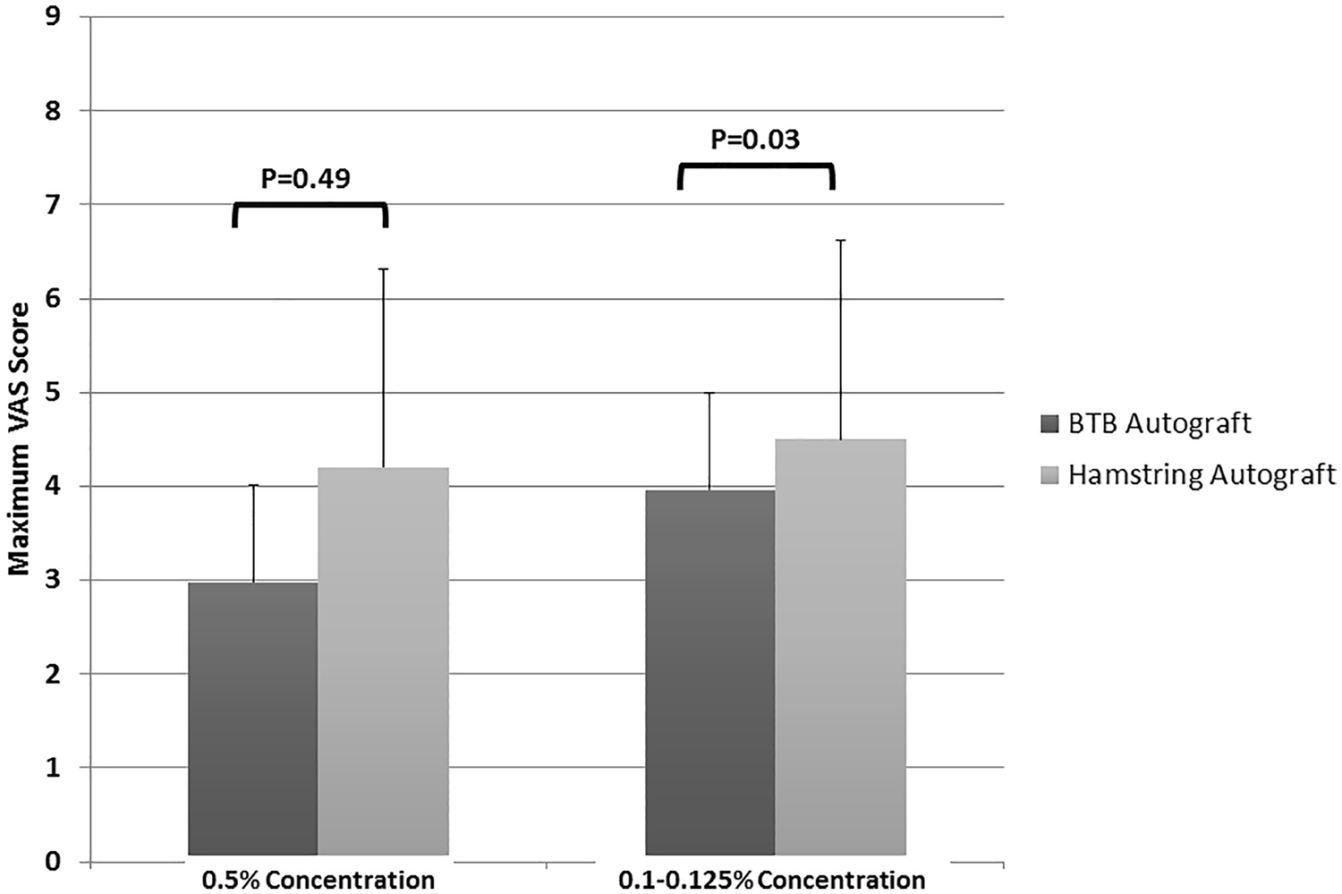
Comparison of maximum postoperative VAS scores of bone patellar bone (BTB) autograft and hamstring ACL reconstructions at different femoral nerve block concentrations. Abbreviations: *ACL *anterior cruciate ligament, *BMI *body mass index, *EMG *electromyography, *FNB *femoral nerve block, *PACU *post-anesthesia care unit, *SD *standard deviation, *VAS *visual analog scale

When comparing only primary ACL reconstruction cases, there were again no significant differences found in maximum VAS (*P* = 0.26), PACU time (*P* = 0.41), or need for an additional postoperative peripheral nerve block (*P* = 0.142). Similar results were found for revision cases for all perioperative factors between groups (*P* > 0.05, respectively). When comparing the type of graft used, patients who underwent hamstring autograft had significantly higher maximum VAS scores (*P* = 0.03) compared to BTB autograft, when a low concentration FNB was used (Fig. 2). Additionally, those with a hamstring autograft harvest were significantly [OR 2.4; 95% CI 1.5 – 4.4] more likely to undergo a postoperative rescue block than patellar tendon autograft (*P* = 0.005), regardless of the FNB concentration (Table 3). A previous history of opioid use was also a significant risk factor for the requirement of a postoperative rescue block (*P* = 0.035).

**Table 3:** Odds ratio for factors predicting the need for postoperative rescue block

Risk factor	Odds Ratio	95% CI	*P*-value
Previous hx of opioid use	6.9	1.1–42.1	0.035*
Hamstring autograft	2.4	1.3–4.4	0.005*
Intraoperative narcotic dose	1	0.99–1.0	0.15
Tourniquet used	0.87	0.44–1.7	0.68
Low Concentration pre-op block	1.77	0.94–3.3	0.07

Abbreviations: *Hx *history, *CI *confidence interval

## Discussion

The most important finding of this study was that using one-fifth the standard local anesthetic concentration during a preoperative femoral nerve block for ACL reconstruction resulted in similar perioperative pain scores, PACU time, need for rescue blockade, and additional immediate opioid requirements, while maintaining adequate quadriceps motor function for a straight leg raise. These results demonstrate the efficacy of a lower concentration FNB to be used for patients undergoing ACL reconstruction.

The delicate balance required to control postoperative pain, without limiting the functional requirements needed for rehabilitation, has lead to multiple modalities of anesthetics being investigated [21]. With previous studies demonstrating postsurgical pain to be the highest within 24 h of the day surgery [9, 11], a single-shot femoral nerve block has been shown to be an effective method for pain control [7, 17, 21]. Veneziano et al. compared three concentrations of local anesthetic for FNB (ropivicaine 0.2%, bupivacaine 0.25%, or ropivicaine 0.5%) in the pediatric population following arthroscopic knee surgery [23]. Unlike the current study, the authors found that the higher concentration local anesthetic resulted in significantly lower immediate postoperative pain scores and shorter PACU time [23]. Considering that the minimal clinically important difference for the VAS score has been reported to be 1.0 in the setting of femoral nerve blocks after ACL reconstruction [13], the mean difference of 0.5 points between the standard- and low-concentration FNB does not seem to be of clinical relevance.

Wulf et al. compared the motor block and pharmakinetics of various local anesthetic concentrations, specifically for those undergoing ACL reconstruction [26]. When using a third of the standard dose of ropivicaine, the authors found no significant difference in postoperative pain, analgesic requirement, or quadriceps paralysis at four hours [26]. Besides, their study did find, as expected, significantly higher plasma levels of the local anesthetic with the higher concentration FNB [26]. However, its effects on the long-term outcomes following ACL reconstruction continue to be poorly understood.

Several thoughts have been proposed regarding the possible causes for longer-term postoperative quadriceps dysfunction. Transient neuropathy and femoral nerve damage have been reported, however, the incidence of these complications has been estimated to be only 2.7% following a single-shot FNB performed with the use of a nerve stimulator [25]. However, this estimate may be under-reported, as demonstrated by Albrecht et al., who obtained electromyography (EMG) testing at 4 weeks and 6 months following ACL reconstruction in patients with a single-shot FNB [1]. The authors found that roughly 25% of patients had clinical and/or EMG evidence of femoral neuropathy at 4 weeks, with nearly all resolving by six months [1]. Thus, it may be assumed that some of the patients of the present study would show clinical and/or EMG evidence of transient femoral neuropathy at later follow-up examinations.

The current study found that hamstring autograft was significantly more likely to require an additional postoperative sciatic nerve block, regardless of local anesthetic concentration used for FNB. This may be explained by increased posterior knee pain during graft harvesting that is not controlled with a femoral nerve block. This was supported by Cappellari et al., who found that significantly less intraoperative analgesic supplementation was required when a posterior psoas compartment block was combined with a 3-in-1 FNB [5]. While our study demonstrated no immediate differences between graft types, longer-term outcomes are needed to identify if patients with quadriceps autograft or additional concomitant procedures could compound the effect of muscle function.

There were several limitations of this study. First, the retrospective design subjects the results to possible selection bias. For instance, the exact reason why each person was assigned a different concentration is not known. Second, this study did not capture functional or isokinetic testing of the quadriceps. Due to the goal of this study being validation of the use of a significantly decreased concentration of local anesthetic for FNB in the immediate postoperative period, long-term functional outcomes were not collected. Third, the influence of surgical technique between three different surgeons may have confounded the results. However, no significant differences in patient demographics, rate of concomitant procedure, or type of graft were identified between groups. Finally, there was no control (patients without a FNB) for comparison.

## Conclusion

Using 1/5th to 1/4th the standard local anesthetic concentration for preoperative femoral nerve block in ACL reconstruction was not found to significantly differ in peri-operative outcomes, PACU time, need for rescue blockade, or additional immediate opioid requirements.
